# Effect of infill ratios in SLA 3D printing on mechanical properties of castable wax patterns for molded shells in investment casting

**DOI:** 10.1371/journal.pone.0311245

**Published:** 2025-02-20

**Authors:** Thanh Tan Nguyen, Van Tron Tran, Van–Thuc Nguyen, Van Thanh Tien Nguyen

**Affiliations:** 1 Faculty of Mechanical Engineering, Ho Chi Minh City University of Technology and Education, Ho Chi Minh City, Vietnam; 2 Faculty of Mechanical Engineering, Industrial University of Ho Chi Minh City, Ho Chi Minh City, Vietnam; Kerman University of Medical Sciences, ISLAMIC REPUBLIC OF IRAN

## Abstract

Investment casting has become an integral part of the modern industry’s manufacturing process with high precision. However, this technology still faces several challenges that need to be addressed for process improvement, especially the complex and flexible part. This research demonstrates the possibility of applying additive manufacturing techniques (3-dimensional printing (3DP)) and castable wax in investment casting. The main objective is to investigate the effect of infill ratios on the mechanical properties of 3D printed patterns and evaluate the ability to create mold shells using the printed patterns for casting stainless steel SUS 304. The results indicate that the infill density considerably influences the printed samples’ mechanical properties, mold-creating ability, weight, and building time. The mechanical properties of the printed samples, including Young’s modulus, tensile strength, and work of extension increase from 13.08 MPa, 393.33 MPa, and 4.25 MJ/m3 to 21.72 MPa, 671.48 MPa, and 9.62 MJ/m3, respectively. Moreover, the infill ratios of printed patterns, less than 25%, can be employed to fabricate the IC mold with exceptional quality. The printed patterns’ average surface roughness (SR) is 2.49 μm, while the average SR of the casted parts is 7.33 μm. The results strongly strengthen the idea of applying the 3DP technique and castable wax substance in investment casting (IC).

## Introduction

Investment casting, or lost wax casting, is a well-established manufacturing technique renowned for its exceptional accuracy and surface finish. It suits diverse industries such as aerospace, automotive, jewelry, and dental [1–6]. The four primary steps of the IC process are pattern creation, shell mold construction, metal pouring, and shell removal from cast parts [7, 8]. Metallic molds prepared using traditional machining methods, such as milling and turning, are usually used to create the wax patterns for IC [6]. However, these techniques face challenges when fabricating design complexity, dimensional accuracy, and high-hardness materials [9]. In addition, the wax pattern is commonly utilized in the IC. Still, its use has certain issues and technical challenges, such as ceramic shell cracking and wax pattern expansion [3].

3D printing, or additive manufacturing, has emerged as a transformative technology with wide-ranging applications in various industries. It offers design freedom, reduced material waste, faster prototyping, shorter production cycles, and driving innovation and sustainability efforts [10, 11]. Recent research has shown that the printing angles and the layer height of the printed part barely influence the mechanical properties of the printed materials for the ranges considered (printing angles are adjusted from 0 to 90 degrees, and the layer height is reduced from 100 μm to 50 μm) [12]. The surface roughness of the printed part in fused deposition modeling (FDM) printers is greater than in Stereolithography (SLA) printers [3, 13]. Additionally, the infill pattern greatly influences the tensile strength of the printed part in FDM printing technology by RepRap printers. The lower the layer thickness, the higher the printed part’s tensile strength and elastic modulus. Concentric infill patterns yield the best elongation, while rectilinear infill patterns have the best strength [14]. The ability to incorporate 3D printing techniques into the IC pattern-making stage is made possible by Additive Manufacturing (AM) technology advancements. It can be casted for extremely thin-walled single crystals [6, 14].

In some research applications of 3DP in investment casting, the result showed that shell molds crack due to the high thermal expansion of thermoset models during burnout [2]. In addition, Nguyen and co-workers [15] showed that the hollow structure could solve the crack in SLA technology with castable wax material. Still, more details about the structure and effect of density on mechanical properties were not mentioned. The 3DP software is also no way to modify the infill structure. The solid structure takes much time and material to print, making mold cracks. So far, little attention has been paid to the mechanical properties of 3DP objects manufactured using an infill pattern to minimize weight, which is a significant advantage of 3D printing over traditional production methods.

To fulfill the gap in technology, the influence of the infill structure ratio of castable wax in SLA printing on the weight and mechanical properties of 3D printed patterns and the ability to create a molded shell for investment casting was investigated. In detail, The relationship between various infill ratios and pattern quality, mechanical performance, and mold shell formation was evaluated. This research aims to provide valuable insights for optimizing the SLA printing process with the hollow structure, enhancing the quality of investment castings.

The scientific contributions of this paper are focused on enhancing the integration of SLA 3D printing in investment casting. First, it assesses the impact of various infill densities on the mechanical properties of SLA 3D printed patterns, which are used for mold-making in casting. It also investigates the correlation between infill density, mass, and printability to optimize material usage and reduce the weight of printed parts. Additionally, the study explores the effect of infill density ratios on mold cracking during heating, addressing a key challenge in preventing defects. Lastly, it evaluates the relationship between the surface roughness of printed parts and the quality of the final metallic cast products, providing insights for improving surface finish and overall product quality. These contributions collectively aim to improve efficiency, material optimization, and quality in the investment casting process.

The structure of this paper is organized as follows: In the next section, we will detail the materials, methods, and preparation involved in this study. Subsequently, we will present and discuss the results to elucidate the findings. Before concluding the investigation, an evaluation of the surface roughness of both 3D printed patterns and cast parts will be provided.

## Materials and methods

### Materials

#### Castable wax resin

Castable Wax resin [16, 17] is supported by Formlabs, Somerville, Massachusetts, United States (imported by 3D Smart Solutions Co., Ltd., Ho Chi Minh City, Vietnam), a specialized photopolymer resin used in Stereolithography (SLA) 3D printers for creating intricate patterns used in investment casting processes, with parameters shown in Table 1 [2, 18].

**Table 1:** Properties of resin castable wax V1.

Parameter	Value
**Ultimate tensile strength**	12 MPa
**Tensile modulus**	220 MPa
**Elongation at break**	13%
**Temperature of 5% mass loss**	249°C
**Ash content (TGA)**	0.0–0.1%

#### Stainless steel SUS 304

Stainless steel SUS 304 is supplied by Dong Luc Joint Stock Company, Binh Duong, Vietnam. The chemical compositions of the 304 austenitic stainless steel are shown in Table 2.

**Table 2:** Chemical composition (wt.%) of the 304 austenitic stainless steel [19].

Element	C	Si	Mn	P	S	Ni	Cr	Fe
**Content**	0.042	0.360	0.550	0.015	0.020	8.601	17.822	Balanced

### Preparation

#### Prepare structure sample for tensile test and 3DP pattern

**Fig 1:**
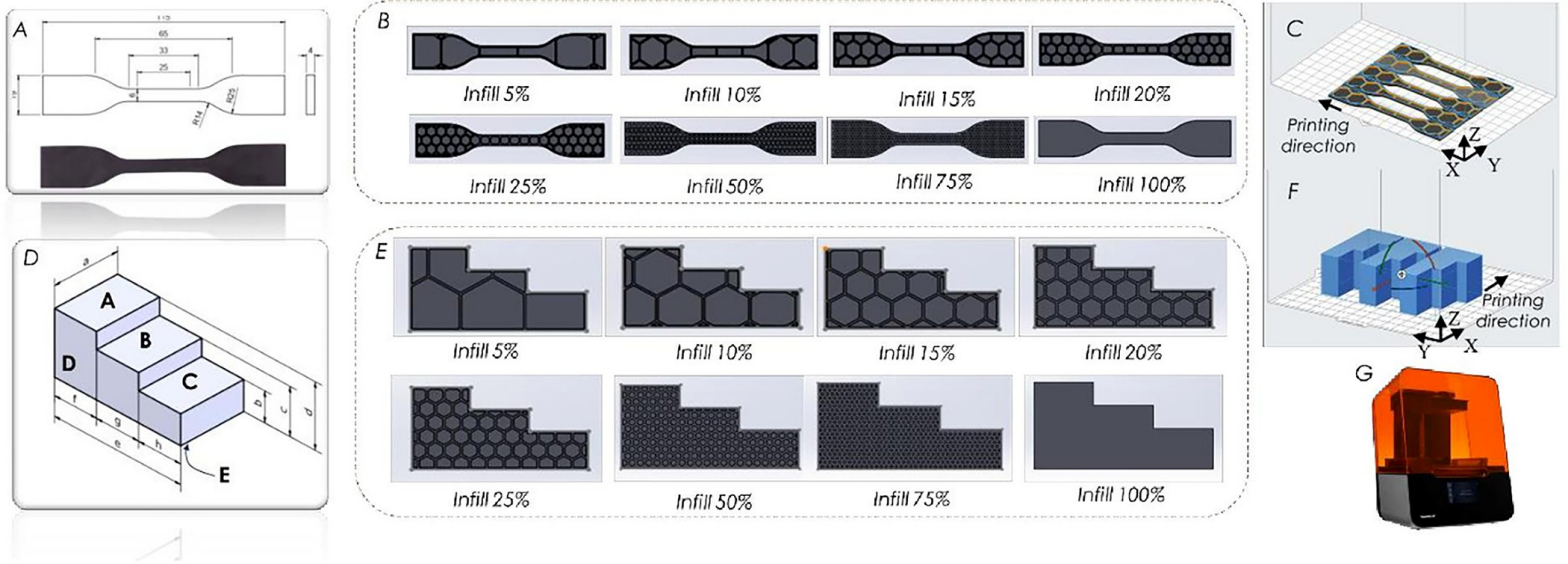
(A) Design of the tensile test sample, (B) tensile test samples with various infill ratios, (C) printing direction tensile test sample, (D) design of surface roughness test sample and measurement position, (E) surface roughness test with difference infill ratio, (F) printing direction step sample pattern, and (G) Form 3 3DP machine.

To evaluate the effect of filling ratios on the mechanical properties of the 3D-printed castable wax materials, a series of tensile testing samples following the ASTM D638—Type IV standard [20, 21] were designed with uniquely honeycomb structures as shown in Fig 1A–1C. The step-type pattern was chosen to survey the effect of the infill density ratio on mass, creating shell-mold ability, and surface roughness of the pattern (Fig 1D–1F).

Infill ratios of samples with 5%, 10%, 15%, 20%, 25%, 50%, 75%, and 100% wt.% were designed using SOLIDWORKS software (Dassault Systèmes, Vélizy-Villacoublay, France). They were converted to STL files using PreForm software (Formlabs, Somerville, MA, USA). Next, the printing parameters were set using the PreForm software, and the obtained files were transferred into Formlabs Form 3 printer (Formlabs, Somerville, MA, USA) for printing (Fig 1G). The printing parameters with a layer thickness of 0.05 mm are set on the machine. The printing direction followed Fig 1C and 1F. It took approximately 3 hours to print each tensile testing sample. After printing, the specimens were washed in isopropyl alcohol (IPA) for about 15 minutes. Subsequently, the samples were dried in the Form Cure machine. They were exposed to UV light for approximately 5 minutes at 50°C.

#### Tensile test

**Fig 2:**
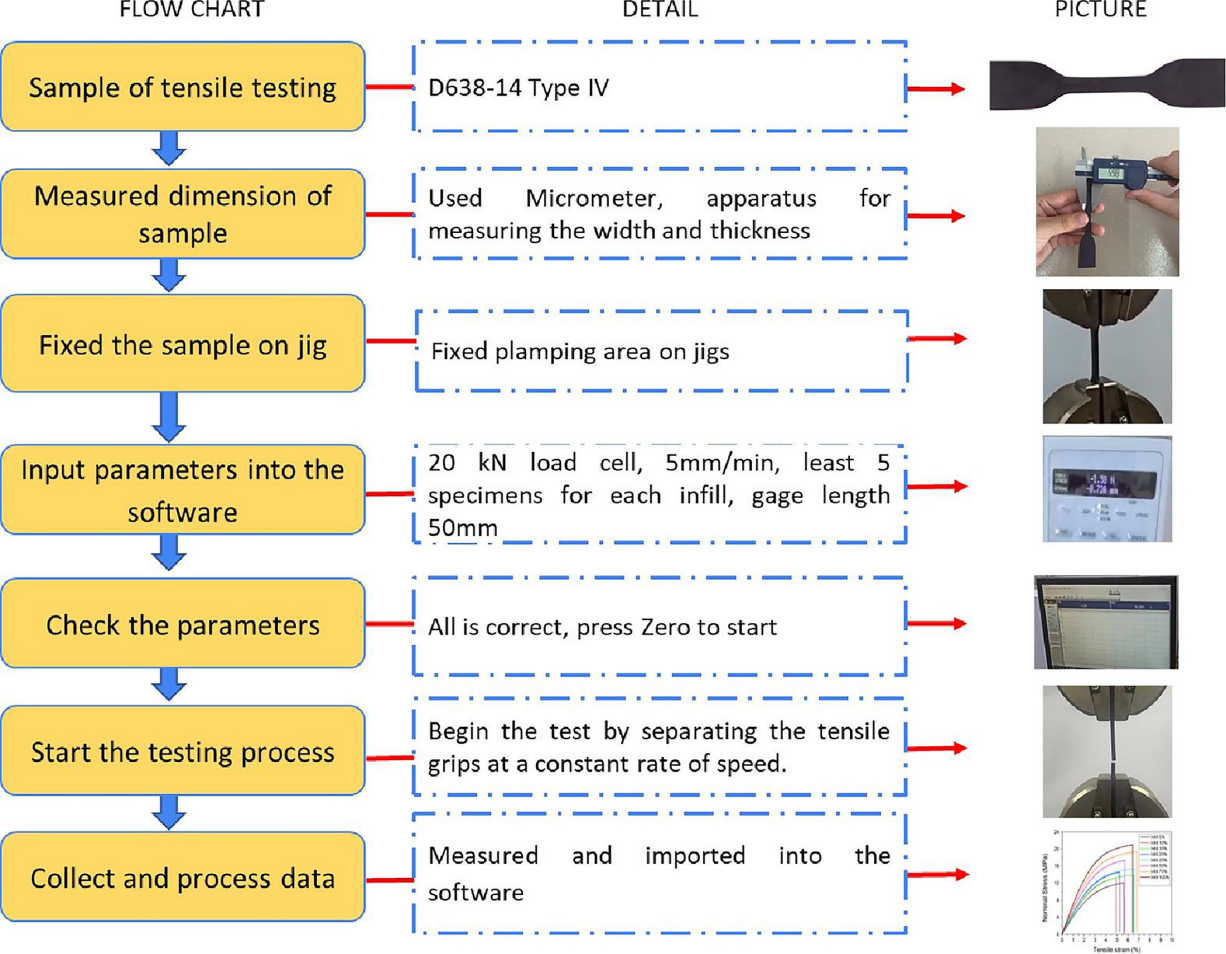
Process of tensile test.

The Shimazu tensile tester (Shimazu Nakagyo, Kyoto, Japan) with a 20-KN load cell was used for the tensile tests. The sample was clamped on the machine with a gauge length of 50 mm. The upper clamp moved upward at 5 mm/min speed until the sample fractured (Fig 2). Each experiment was repeated 5 times. The error was calculated using the mean deviation method [22].

#### Preparation of shell mold

**Fig 3:**
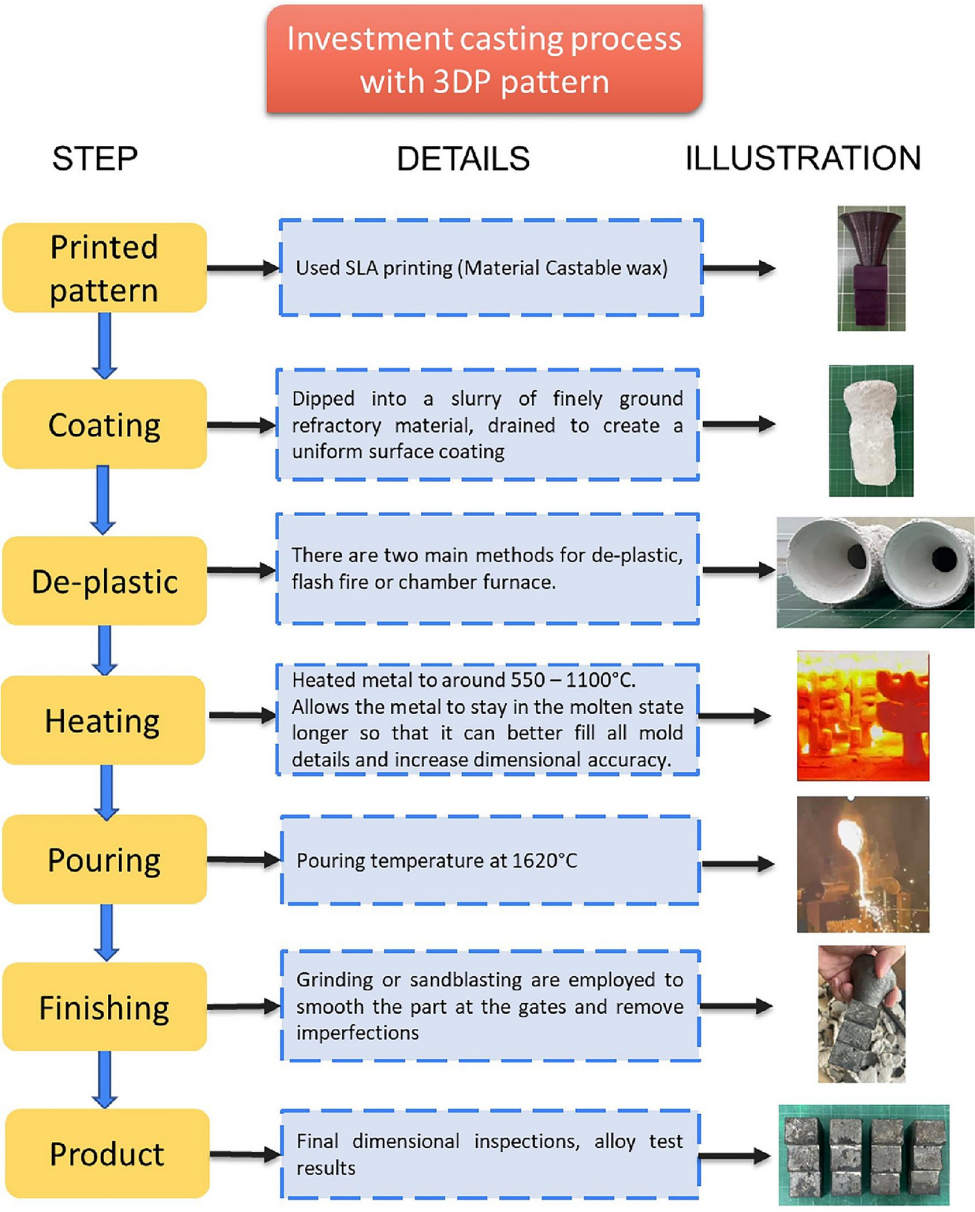
Investment casting process with 3DP pattern.

The printed patterns were coated four times with ceramic as the following step: dipped in first solution (including colloidal silica 830 consisting of 16.86 wt%, zircon flour 83.3 wt%, de-foaming 0.1 wt%, and degassing 0.06 wt%), second solution (include colloidal silica 1430 26 wt%, Mullite flour 200F 54 wt%, distilled water 20 wt%), back solution (include colloidal silica 1430 24 wt%, Mullite flour 200F 68 wt%, distilled water 8 wt%), and Last solution (Mullite flour 200F 34 wt%, + A105 flour 26 wt%, + silica 32,6 wt%+ distilled water 7.4 wt%). After being coated, the 3DP patterns were removed by heating to obtain the molds (1100°C) Then, the molten metal was poured into the molds through a gate system at 1620°C. After the molten metal completely solidified, the molds were knocked out, and the cast parts were removed. Subsequently, the gating system was removed. Finally, the desired casted parts were obtained, and their surface roughness was tested (Fig 3).

**Fig 4:**
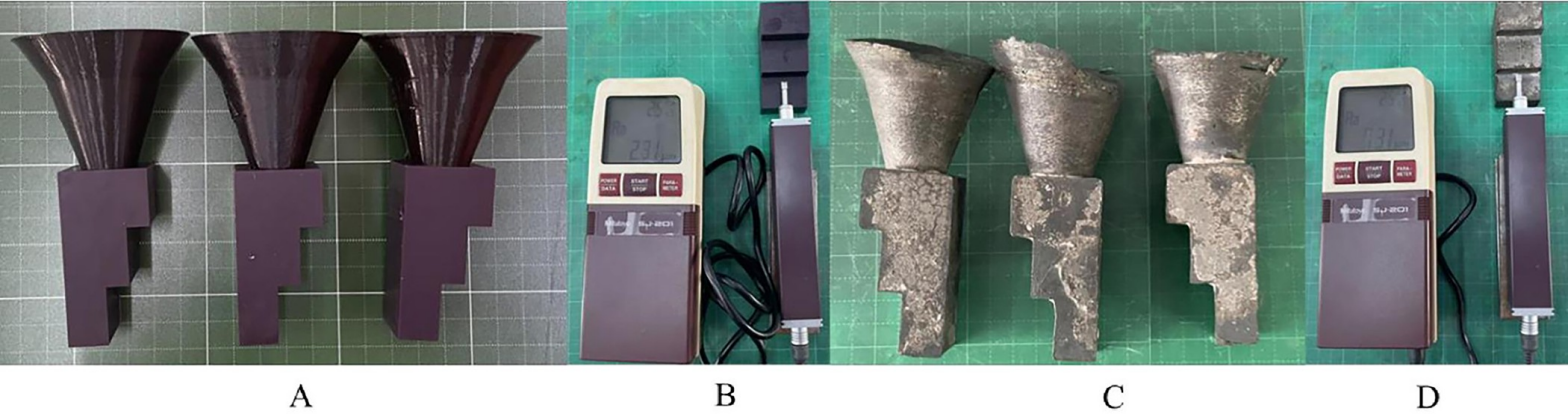
(A) 3D printed samples, (B) roughness testing of 3D printed samples, (C) casted part samples, and (D) roughness testing of casted parts.

**Roughness test.** Mitutoyo SJ–201 roughness tester (Mitutoyo, Kawasaki, Kanagawa, Japan) was used to measure the surface roughness of the 3D printed part and cast part (Fig 4A–4D). A roughness test based on the B46.1–2019 standard, in which the cut-off length of 0.8mm was employed for testing the SR (Ra) in the range of 0.1–2 μm, while the cut-off length of 2.5 mm was used for determining the SR (Ra) in a range of 2–10 μm. For each specimen, three measurements were carried out.

## Results and discussion

### Effect of infill ratio on mechanical properties of castable wax printed parts

**Fig 5:**
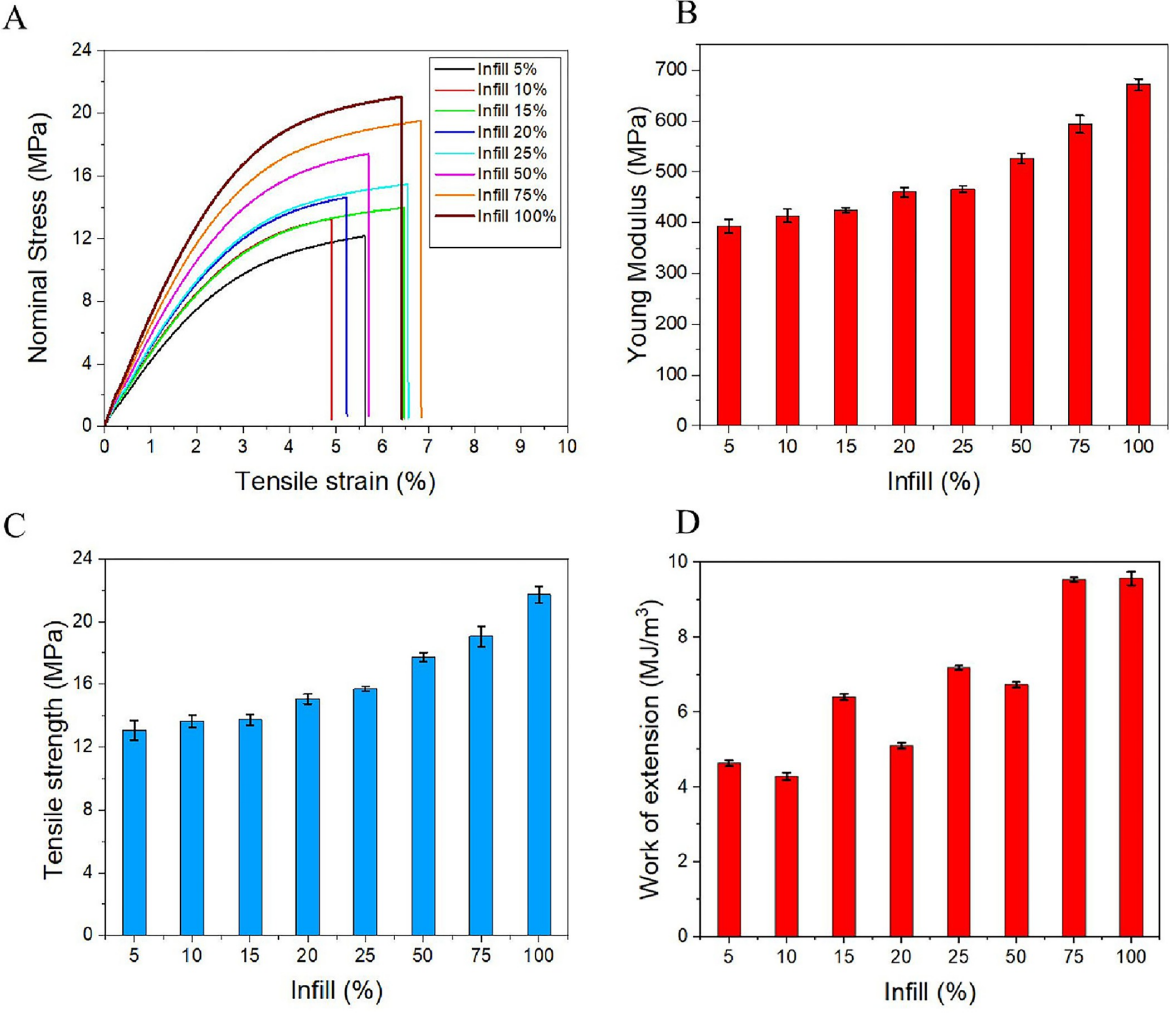
(A) Tensile stress-strain curves, (B) Young’s modulus, (C) tensile strength, and (D) work of extension of the various printed specimens with increased infill ratios. In plots (B)–(D), error bars indicate the mean absolute deviation (n = 5).

This investigation aims to determine the infill ratio’s effect on the mechanical properties of the printed samples. Fig 5 displays the mechanical properties of the printed wax with different infill ratios. The results indicated that an increase in the infill ratio led to an increase in the mechanical properties of the printed parts. Young’s modulus and tensile strength of the printed samples increased from 393.33 MPa to 671.48 MPa and 13.08 MPa to 21.72 MPa respectively (Fig 5A–5C). The work of extension increased from 4.25 to 9.62 MJ/m^3^ (Fig 5D). Our finding is consistent with recent studies, which reported that the tensile strength of castable wax structures varies from 11.6 MPa to 23.13 MPa [23]. When the infill percentage is increased, the number of connecting lines between layers increases, creating a larger contact area. This enhances the bonding strength between layers and increases the overall strength of the object. With a greater amount of fill material, the cross-sectional area of the material increases, making the part more rigid and less prone to deformation under load. A high infill percentage also minimizes internal voids, reducing the likelihood of crack formation and contributing to increased strength. This result differs somewhat from the raw material properties of the supplier in Table 1. Thus, the mechanical properties of a 3D-printed part depend not only on the material but also on the infill percentage, infill pattern, and printing parameters.

### Effected of infill ratio on the weight of the 3D printed patterns

**Fig 6:**
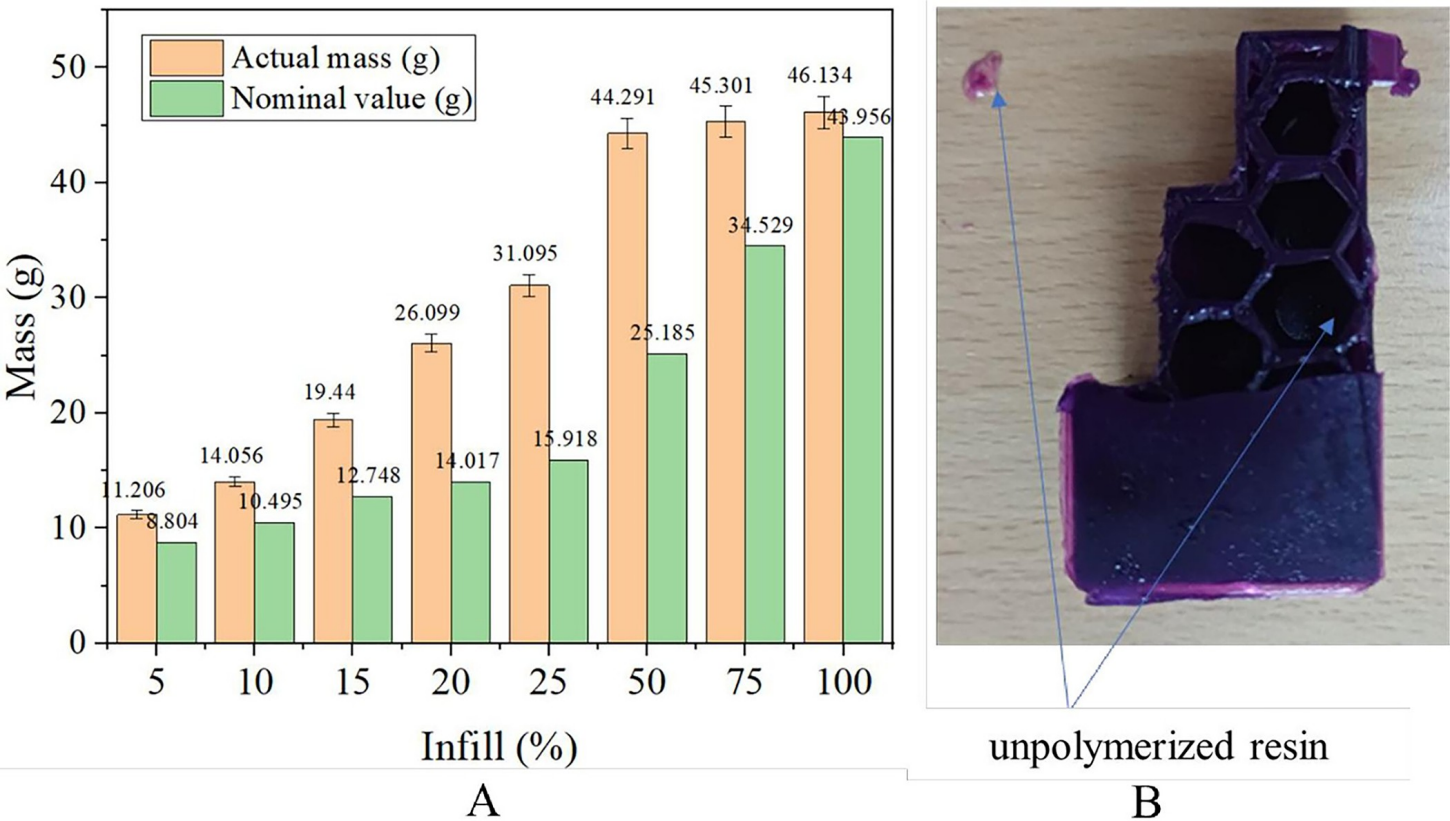
(A) Compare actual and nominal mass values. (B) Unpolymerized resin.

Castable wax was designed to burn out cleanly without leaving any residue, and smooth surfaces produce high-quality final products. With its exceptional properties, Formlabs Castable Wax resin was chosen to create intricate, high-quality products. Manufacturers can confidently rely on this material to produce accurate and reliable casts, achieving excellent results in their manufacturing processes. Castable wax with urethane dimethacrylate 60–80%, photoinitiator <1.5% [19]. With a 20% wax fill, castable wax resin can be applied in IC casting without ash and with clean melting [22]. According to previous reports [16, 23–27], among several infill structures used in 3D printing, including hexagonal (honeycomb), rectilinear, triangular, concentric, gyroid, rhombic, spherical, solid, wiggle, and grid structures, the honeycomb infill structure pattern shows a high elastic modulus and tensile strength compared to others. Therefore, this structure was employed in this study. The weight of the designed patterns was calculated using the SOLIDWORKS software, while an electronic scale measured the weight of the printed ones. The obtained results are shown in Fig 6A. The designed pattern’s weight was relatively higher than that of the designed patterns at all infill ratios (Fig 6A). This was due to unpolymerized resin entrapped inside the space of the structure or remaining in the outer printed surfaces (Fig 6B). The designed and printed pattern weights generally increased as the infill density increased from 5% to 100%. In particular, the 5% infill sample weighed 11.206 g, while the 100% infill weighed 46.134 g. This indicated more material was used as the infill density became denser, increasing the pattern weight. The designed pattern’s weight slightly increased from 8.804 g at a 5% infill ratio to 15.918 g at a 25% infill ratio (approximately 0.356 g per percent). With infill increasing from 25% to 100%, the result showed a larger increase (approximately 0.373 g per percent), from 15.918 g to 43.956 g. However, the weight of the printed part increased irregularly by around 0.994 g per percent from 5% to 25% infill ratio and around 0.2 g per percent from 25% to 100% infill ratio. Lower infill ratios (from 5% to 25%) resulted in a low-density infill material inside the 3D-printed object, providing more space for the entrapping of the resin during printing (Fig 6B). Unpolymerized resin could become trapped in the model during the printing and curing process, SLA slicing software does not allow for the adjustment of infill. As a result, the hollow structure increases resin entrapment inside, thereby causing a deviation in the actual mass from the theoretically calculated mass.

### Effected of infill ratio on the ability to create mold shell

**Fig 7:**
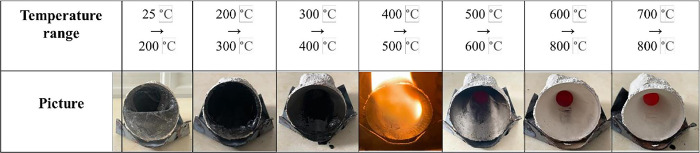
The de-plastic process with various temperatures.

**Fig 8:**
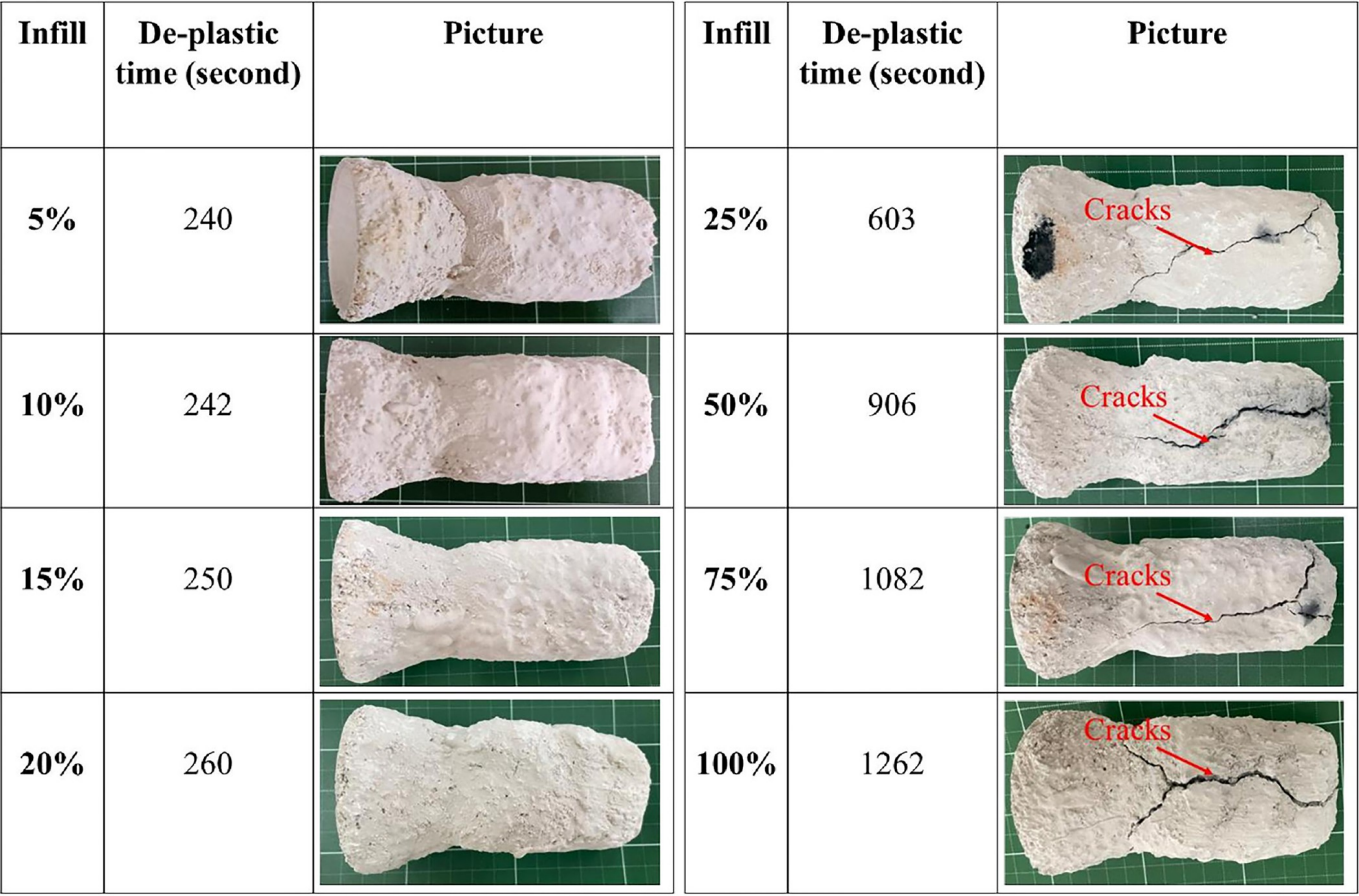
The de-plastic process done under the same temperature conditions and different infill ratios.

This experiment aimed to determine the ability to create mold shells with different infill ratios. Following the completion of dimensional measurements, the printed parts were utilized to create a molded shell. The sample was heated from room temperature to 800°C; the result is shown in Fig 7; the patterns were plasticized at 200°C, melted at 300°C, burnt at 500°C, and completely burnt out at 700°C. The transformation of the 3D-printed sample during sintering is illustrated in Fig 7. The more the infill ratio increased, the more de-plastic time was consumed (Fig 8).

**Fig 9:**
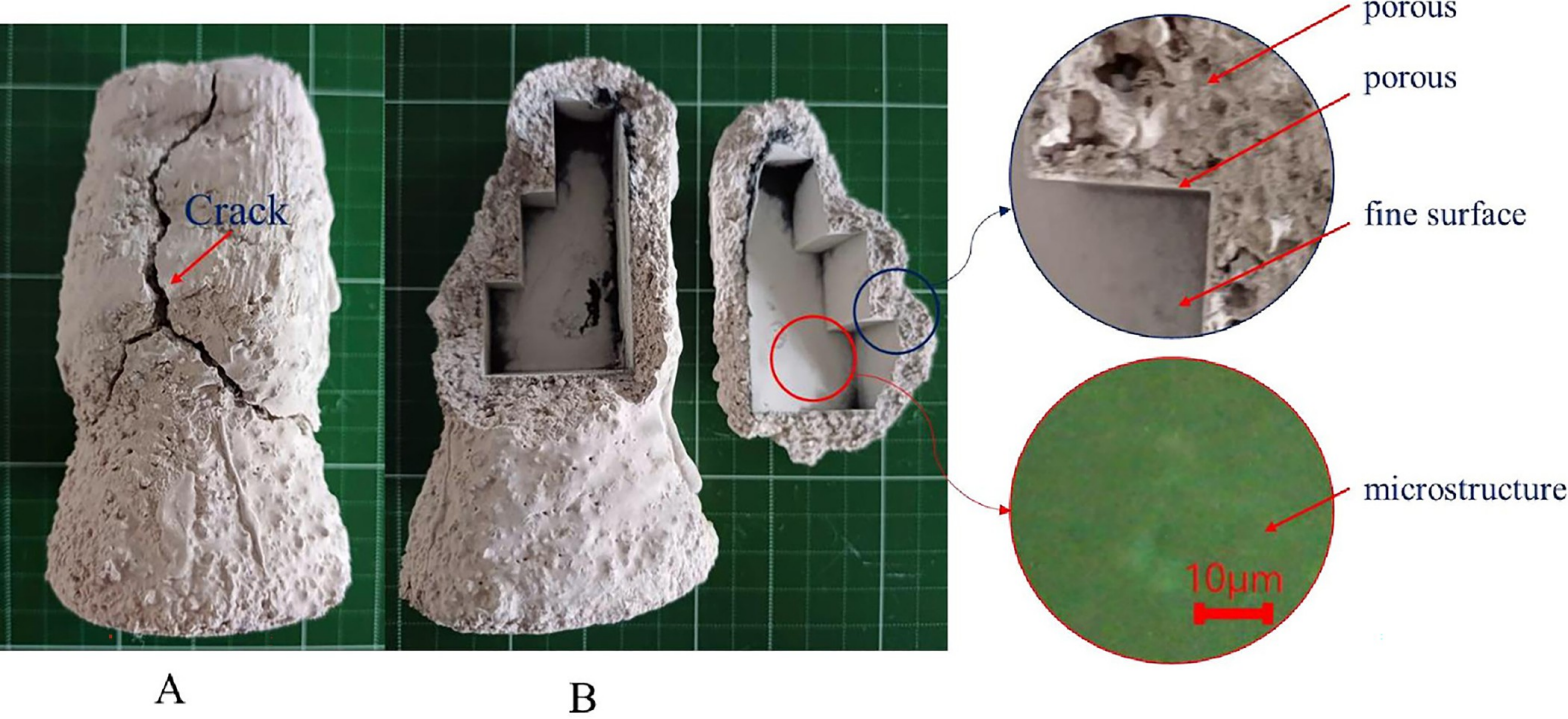
(A) Mold with cracks, (B) inside crack with porous and surface microstructure.

The effect of the infill density of the wax patterns on the burning time was also evaluated in this study. The results indicated that with low infill densities (ranging from 5% to 20%), the time to burn the resin completely was approximately 4 minutes (240–260 s). In comparison, for 100% infill density, the required time was around 21 minutes (1262 s). Moreover, the infill density significantly influenced the mold shell quality. With the low infill densities (below 25%), the mold shell was of good quality without any visible cracks. However, as the infill density increases beyond 25%, cracks appear on the mold shell after the de-plastic process. (Fig 9A and 9B). This problem was due to the thermal expansion of castable wax material higher than ceramic shell mold [28, 29]. Moreover, cracks happened due to several issues, such as thermal shock at high temperatures during the burnout process [3, 29, 30], micro-cracks formed in the shell mold during the fabrication process [2], and an increase in pressure in the closed pores in the shell mold cracks [30]. When the pattern’s expansion coefficient exceeds the shell’s coefficient of expansion, the shell fractures [29]. This expansion of the wax pattern strains the ceramic mold, causing mold cracks that could cause mold damage or expensive repairs [4]. Additionally, because of a discrepancy in thermal expansion coefficients, the existence of many interfaces in the mold due to functional grading may cause residual stresses to arise during cooling. This frequently results in the initiation and spread of interfacial cracks, which weaken the mold’s mechanical strength and cause the layered structure to delaminate [1]. Thus, the printed patterns’ rational infill densities for obtaining the mold shell’s high quality were less than 20% (Fig 8). When metal is cast at high temperatures, surface imperfections (blow holes) and porosity are always present due to issues, including shell cracking and the permeability of the ceramic mold [3]. Therefore, it is essential to carefully select the pattern material and its internal structure to ensure that its thermal expansion coefficient matches the selected ceramic material; if not, this would result in significant thermal stresses during firing that will eventually cause breaking in the ceramic shell [5].

A porous construction can effectively solve the issue of shell cracking. Cost and time reductions would result from reducing material costs and manufacturing time required to create patterns. Nevertheless, whether or not this approach works for large and complex castings needs more research. Combining two or three techniques may be necessary to stop the shell from cracking in complex and large castings [29].

### Evaluate the surface roughness of 3DP patterns and cast parts

**Fig 10:**
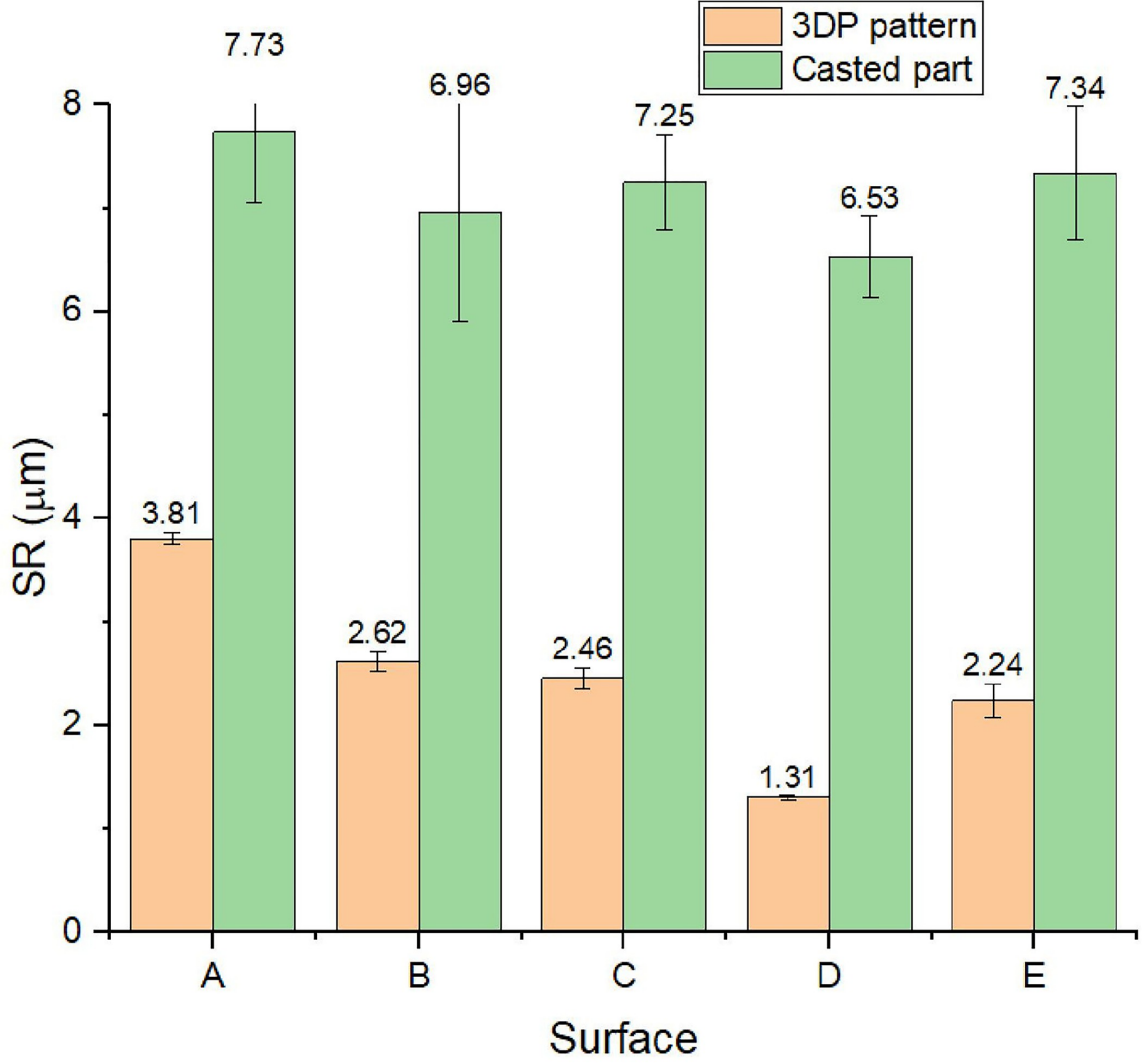
The surface roughness of the 3D printed parts and cast part (R_a_ in micrometer).

To evaluate the surface roughness (SR) of the 3DP patterns and casted parts, the roughness values at surfaces A, B, C, D, and E surfaces were tested (Fig 1C). The printed patterns generally had an average SR of 2.49μm, and the casted parts had an average SR of 7.33 μm. Surface D exhibited the lowest SR (Ra) (1.31μm) [31]. The surfaces A, B, C, and E displayed SR (Ra) values of 3.81μm, 2.62μm, 2.46μm, and 2.24μm, respectively. It was found that the printed pattern’s surface affected the casted part’s SR. As shown in Fig 10, the SR of the casted parts consistently varied following the SR of the printed patterns. For instance, among these measured positions, surface D had the lowest roughness (Ra = 6.53 μm), while The obtained values at surfaces A, B, C, and E ranged from 6.96 μm to 7.73 μm. The obtained SR of the casted parts in this study is comparable to those in previous studies, in which different methods created the casted parts. For example, the surface roughness of the FDM-based ABS patterns is 14.40 μm [32] The result of the SR wax pattern was similar to that reported in the recent study [33] Ra = 3.2 μm, [34] (0.8–1.8 μm), [35] (1.3–2.4 μm), and [36] (1.02–2.83 μm). This research demonstrates that SLA 3D printing offers high precision, smooth surfaces, and high tensile strength. However, it presents challenges in producing intricate hollow structures owing to the need for support materials and layer thickness constraints. The findings provide a practical framework for designing hollow structures from a 3DP pattern to prevent mold cracking during the investment casting process.

## Conclusions

The research has shown that the hollow structure effectively minimizes cracking during the molding process while also reducing the weight and printing time of the sample. This study provided a foundation for more in-depth research on hollow structures in SLA printing, thus facilitating the design of 3D SLA printed structures. Based on the results, this study can be conclusions:

- The higher infill ratios lead to an improvement in the mechanical properties of 3DP patterns. In particular, Young’s modulus and tensile strength vary from 393.33 MPa and 13.08 MPa to 671.48 MPa and 21.72 MPa, respectively.
- The infill ratios of the printed patterns are higher than 25%, resulting in cracks in the mold shell due to the expansion of the printed part material. Infill ratios ranging from 5% to 20% are recommended for preparing the IC printed patterns.
- The increase in the infill ratio results in an increase in the mass of the printed part. However, the actual weight is heavier than the nominal weight because the resin is trapped inside the structure.
- The surface roughness of the casted part is higher than that of the 3D-printed part. The printed patterns have an average SR of 2.49 μm, and the casted parts have an average SR of 7.33 μm. The highest quality surface with SR (Ra) of 1.31 μm is obtained in the XY plane.

The findings results strongly support the idea of using the 3DP technique in investment casting (IC). However, further studies investigating the effects of mold wall thickness on crack resistance and the influence of internal structure on the printed parts’ mechanical properties and their expandability are necessary.
